# What Not To Do to Thrive in your Career?

**DOI:** 10.1192/j.eurpsy.2022.200

**Published:** 2022-09-01

**Authors:** N. Sartorius

**Affiliations:** Association for the Improvement of Mental health Programs (AMH), President, Geneva, Switzerland

## Abstract

What not to do to thrive in your career The presentation will examine options that are usually available to psychiatrists during their training and early in their career and propose criteria which should be used to select or discard them. Among the criteria proposed – in addition to personal interest - should be the amount of time that engaging in a particular pursuit might take, the potential gain of the engagement later on in one’s career, the likelihood of expanding the circle of friends and acquaintances and several others.

**Disclosure:**

No significant relationships.

